# Palm oil protects α-linolenic acid from rumen biohydrogenation and muscle oxidation in cashmere goat kids

**DOI:** 10.1186/s40104-020-00502-w

**Published:** 2020-10-05

**Authors:** Xue Wang, Graeme B. Martin, Qi Wen, Shulin Liu, Yinhao Li, Binlin Shi, Xiaoyu Guo, Yanli Zhao, Yangdong Guo, Sumei Yan

**Affiliations:** 1 Inner Mongolia Key Laboratory of Animal Nutrition and Feed Science, College of Animal Science, Inner Mongolia Agricultural University, Hohhot, 010018 China; 2 Beijing Advanced Innovation Center for Food Nutrition and Human Health, College of Horticulture, China Agricultural University, Beijing, 100193 China; 3 UWA Institute of Agriculture, The University of Western Australia, 35 Stirling Highway, Crawley, WA 6009 Australia

**Keywords:** Desaturases, Docosahexaenoic acid, Hydrogenation, Goats, Meat, Oleic acid, Oxidative stress

## Abstract

**Background:**

In ruminants, dietary C18:3n-3 can be lost through biohydrogenation in the rumen; and C18:3n-3 that by-passes the rumen still can be lost through oxidation in muscle, theoretically reducing the deposition of C18:3n-3, the substrate for synthesis of poly-unsaturated fatty acids (n-3 LCPUFA) in muscle. *In vitro* studies have shown that rumen hydrogenation of C18:3n-3 is reduced by supplementation with palm oil (rich in *cis-*9 C18:1). In addition, in hepatocytes, studies with neonatal rats have shown that *cis-*9 C18:1 inhibits the oxidation of C18:3n-3. It therefore seems likely that palm oil could reduce both rumen biohydrogenation of C18:3n-3 and muscle oxidation of C18:3n-3. The present experiment tested whether the addition of palm oil to a linseed oil supplement for goat kids would prevent the losses of C18:3n-3 and thus improve the FA composition in two muscles, *Longissimus dorsi* and *Biceps femoris*. To investigate the processes involved, we studied the rumen bacterial communities and measured the mRNA expression of genes related to lipid metabolism in *Longissimus dorsi*. Sixty 4-month-old castrated male Albas white cashmere kids were randomly allocated among three dietary treatments. All three diets contained the same ingredients in the same proportions, but differed in their fat additives: palm oil (PMO), linseed oil (LSO) or mixed oil (MIX; 2 parts linseed oil plus 1 part palm oil on a weight basis).

**Results:**

Compared with the LSO diet, the MIX diet decreased the relative abuandance of *Pseudobutyrivibrio*, a bacterial species that is positively related to the proportional loss rate of dietary C18:3n-3 and that has been reported to generate the ATP required for biohydrogenation (reflecting a decrease in the abundance of rumen bacteria that hydrogenate C18:3n-3 in MIX kids). In muscle, the MIX diet increased concentrations of C18:3n-3, C20:5n-3, C22:6n-3, and n-3 LCPUFA, and thus decreased the n-6/n-3 ratio; decreased the mRNA expression of *CPT1β* (a gene associated with fatty acid oxidation) and increased the mRNA expression of *FADS1* and *FADS2* (genes associated with n-3 LCPUFA synthesis), compared with the LSO diet. Interestingly, compared to *Longissimus dorsi*, *Biceps femoris* had greater concentrations of PUFA, greater ratios of unsaturated fatty acids/saturated fatty acids (U/S), and poly-unsaturated fatty acids/saturated fatty acids (P/S), but a lesser concentration of saturated fatty acids (SFA).

**Conclusions:**

In cashmere goat kids, a combination of linseed and palm oils in the diet increases the muscle concentration of n-3 LCPUFA, apparently by decreasing the relative abundance of rumen bacteria that are positively related to the proportional loss rate of dietary C18:3n-3, by inhibiting mRNA expression of genes related to C18:3n-3 oxidation in muscle, and by up-regulating mRNA expression of genes related to n-3 LCPUFA synthesis in muscle, especially in *Longissimus dorsi*.

## Background

The long chain n-3 poly-unsaturated fatty acids (n-3 LCPUFA), such as C20:5n-3 and C22:6n-3, have a wide range of biological effects that have long been believed to be beneficial for human health [1, 2]. An important dietary source of n-3 LCPUFA is meat from ruminants, so there have been many attempts to increase the muscle concentration of n-3 LCPUFA in livestock by feeding dietary supplements [3]. In theory, this outcome could be accomplished by feeding the animals with linseed oil because it is rich in C18:3n-3, the substrate for n-3 LCPUFA synthesis [4]. However, dietary C18:3n-3 can be hydrogenated extensively by rumen bacteria [5], potentially leading to a reduction in post-ruminal C18:3n-3 flow. This problem might be overcome by feeding oils rich in *cis*-9 C18:1, as revealed by an *in vitro* rumen study in which palm oil was added to a basal diet – after 24 h incubation, it decreased C18:3n-3 biohydrogenation but enhanced *cis*-9 C18:1 biohydrogenation, in a dose-responsive manner [6]. However, the ruminal microbes involved in this process have not been identified.

The next challenge is that the ruminal “by-pass” C18:3n-3 can be oxidized in the various tissues and organs that are involved in fatty acid (FA) metabolism [7], leading to a reduction in the tissue deposition of C18:3n-3, and therefore the local synthesis of n-3 LCPUFA in muscle.

On the other hand, susceptibility to oxidative stress in mammals is increased by consumption of dietary n-3 PUFA [8, 9]. In the liver, oxidative stress can reduce the gene expression and catalytic activity of Δ-5 and Δ-6 desaturases (*FADS1* and *FADS2*), the most relevant enzymes in n-3 LCPUFA biosynthesis, thereby reducing the tissue levels of n-3 LCPUFA [10]. Again, *in vitro* studies have presented a possible solution – in a study with rat hepatocytes, the oxidative stability of C18:3n-3 was improved by blending C18:3n-3 with *cis*-9 C18:1 [11].

Together, these observations suggest that more substrate and desaturases would become available for the synthesis of n-3 LCPUFA *in vivo* if the biohydrogenation and oxidation of C18:3n-3 could be reduced by blending linseed oil with palm oil in ruminant diets. Moreover, any changes in biohydrogenation pathways in the rumen would probably be explained by changes in the bacterial communities [5]. Therefore, using cashmere kids, we tested whether diets supplemented with a blend of linseed and palm oils increases the muscle concentration of n-3 LCPUFA more than linseed oil alone, and whether this outcome is mediated by i) a reduction in the abundance of bacteria that hydrogenate C18:3n-3 in the rumen; ii) a decrease in mRNA expression of *CPT1β* (a gene related to FA oxidation), and iii) an up-regulation of the mRNA expression of *FADS1* and *FADS2* in muscle.

## Methods

### Animals, diets and feeding management

This study was conducted on the Inner Mongolia White Cashmere Goat Breeding Farm, Wulan Town, Etuoke Banner, Ordos City, Inner Mongolia Autonomous Region, China (39°12′N; 107°97′E). Sixty 4-month-old castrated male kids (average body weight 18.6 ± 0.1 kg) were selected and randomly allocated among three groups, each of which comprised four units of five kids. All three diets contained the same ingredients in the same proportions, but their fat additives differed (Table 1): palm oil (PMO), linseed oil (LSO) or mixed oil (MIX; linseed oil blended with palm oil in a 2:1 ratio based on weight, providing 4.7% fat, 35% C18:3n-3 and 20% *cis-*9 C18:1). For blending, palm oil (Jiali, Shanghai, China) and linseed oil (Mengyue Xiang Biotechnology Co., Ltd., Inner Mongolia, China) were removed from frozen storage and placed in direct sunlight (about 25 °C) to defrost, then mixed in a stainless steel vessel.

**Table 1:** Composition and analysis of experimental diets fed to cashmere goat kids

Item	Day 1–30			Day 31–60			Day 61–90		
	PMO	LSO	MIX	PMO	LSO	MIX	PMO	LSO	MIX
Ingredient (% air dry basis)									
Alfalfa hay particles	25.00	25.00	25.00	15.00	15.00	15.00	12.50	12.50	12.50
Maize straw particles	5.00	5.00	5.00	20.00	20.00	20.00	25.00	25.00	25.00
Tall oat grass particles	20.00	20.00	20.00	15.00	15.00	15.00	12.50	12.50	12.50
Corn	23.37	23.37	23.37	30.40	30.40	30.40	29.90	29.90	29.90
Soybean meal	10.50	10.50	10.50	11.40	11.40	11.40	10.40	10.40	10.40
Distillers dried grains with solubles	7.24	7.24	7.24	0.50	0.50	0.50	0.50	0.50	0.50
Flax cake	4.80	4.80	4.80	3.50	3.50	3.50	4.50	4.50	4.50
Linseed oil	0.00	2.00	1.33	0.00	2.00	1.33	0.00	2.50	1.67
Palm oil	2.00	0.00	0.67	2.00	0.00	0.67	2.50	0.00	0.83
Premix^a^	0.50	0.50	0.50	0.50	0.50	0.50	0.50	0.50	0.50
Calcium carbonate	0.20	0.20	0.20	0.20	0.20	0.20	0.20	0.20	0.20
CaHPO_4_	0.20	0.20	0.20	0.20	0.20	0.20	0.20	0.20	0.20
Sodium chloride	0.54	0.54	0.54	0.50	0.50	0.50	0.50	0.50	0.50
Sodium bicarbonate	0.35	0.35	0.35	0.80	0.80	0.80	0.80	0.80	0.80
Magnesium oxide	0.30	0.30	0.30	0.00	0.00	0.00	0.00	0.00	0.00
Chemical composition									
Digestible energy^b^, MJ/kg DM	12.9	13.1	13.0	12.8	13.0	12.9	12.8	13.1	13.0
CP, g/kg DM	188.2	188.1	188.1	158.8	158.7	158.8	151.5	151.4	151.4
Ether extract, g/kg DM	54.1	51.8	52.6	46.0	43.7	44.4	49.1	46.3	47.2
NDF, g/kg DM	430.3	431.2	431.1	427.6	427.4	427.5	436.3	436.1	436.1
ADF, g/kg DM	230.7	231.7	231.0	235.2	235.3	235.2	240.5	240.3	240.2
Calcium, g/kg DM	11.1	11.1	11.0	10.8	10.9	10.8	10.7	10.7	10.7
Phosphorus, g/kg DM	4.7	4.7	4.7	4.5	4.4	4.5	4.3	4.2	4.3
Fatty acids (% of total)^c^									
C16:0	26.69	10.98	16.25	25.88	9.28	14.77	26.88	8.50	14.61
C16:1	0.50	0.49	0.49	0.51	0.50	0.50	0.49	0.48	0.48
C18:0	2.64	3.18	3.00	2.52	3.10	2.91	2.47	3.10	2.89
*trans*-9 C18:1	0.39	0.39	0.39	0.38	0.39	0.39	0.37	0.38	0.37
*cis-*9 C18:1	29.29	15.19	19.92	29.22	14.33	19.25	31.04	14.55	20.04
C18:2n-6*t*	0.46	0.26	0.33	0.44	0.23	0.30	0.42	0.19	0.27
C18:2n-6*c*	19.22	20.65	20.17	22.53	24.04	23.54	21.06	22.74	22.18
C18:3n-3	8.58	35.59	26.53	6.40	34.91	25.50	5.73	37.30	26.81
C20:5n-3	0.35	0.35	0.35	0.36	0.36	0.36	0.31	0.31	0.31
C22:6n-3	0.66	0.36	0.46	0.73	0.42	0.52	0.73	0.38	0.50
Saturated fatty acids	35.99	20.30	25.57	35.09	18.52	24.00	35.73	17.38	23.48
Monounaturated fatty acids	32.10	19.27	23.57	32.06	18.51	22.99	33.77	18.77	23.75
Polyunsaturated fatty acids	32.08	60.43	50.92	33.03	62.97	53.08	30.71	63.86	52.84

^a^ Provided per kg of premix: iron (Fe) 4 g, copper (Cu) 0.8 g, zinc (Zn) 5 g, manganese (Mn) 3 g, iodine (I) 30 mg, selenium (Se) 30 mg, cobalt (Co) 25 mg, vitamin A (VA) 600,000 IU, vitamin D (VD_3_) 250,000 IU, vitamin E (VE) 1,250 IU, vitamin K (VK_3_) 180 mg, vitamin B_1_ (VB_1_) 35 mg, vitamin B_2_ (VB_2_) 850 mg, vitamin B_6_ (VB_6_) 90 mg, nicotinic acid 2,200 mg, *D*-pantothenic acid 1,700 mg, vitamin B_12_ (VB_12_) 3 mg, biotin 14 mg, folic acid 150 mg

^b^ Digestible energy is calculated based on the ingredients of the diet and their digestible energy content, not based on the actual dry matter intake

^c^ Total fatty acids = saturated fatty acids (6:0 + 8:0 + 10:0 + 11:0 + 12:0 + 13:0 + 14:0 + 15:0 + 16:0 + 17:0 + 18:0 + 20:0 + 21:0 + 22:0 + 23:0 + 24:0) + monounsaturated fatty acids (14:1 + 15:1 + 16:1 + 17:1 + *trans*-9 18:1 + *cis*-9 18:1 + 20:1 + 22:1 + 24:1) + polyunsaturated fatty acids (18:2n-6*t* + 18:2n-6*c* + 18:3n-6 + 20:2n-6 + 20:3n-6 + 20:4n-6 + 22:2n-6 + 18:3n-3 + 20:3n-3 + 20:5n-3 + 22:6n-3)

*PMO* palm oil diet, *LSO* linseed oil diet, *MIX* mixed oil diet

The diets were prepared by manually blending the oil thoroughly into the ground concentrate to ensure homogenous distribution throughout the ration. The diets were prepared fresh twice each day and were offered as a total mixed ration (TMR) in two equal meals at 08:30 and 16:30 h. The kids were given free access to drinking water. The diets were fed for 104 d, consisting of 14 d for adaptation followed by 90 d of treatment. The treatment period was divided into early (1–30 d), middle (31–60 d) and late periods (61–90 d) so the amount and composition of diet offered could be increased to meet the needs of cashmere kids as they grow, according to the feeding standard of meat-producing sheep and goats (China, NY/T816, 2004 [12]; Table 1).

### Sampling and slaughtering procedures

To estimate dry matter intake for five kids in each pen, refusals were collected and weighed 30 min before each feeding, at 08:00 h daily. After weighing, the refusals were evenly sprinkled on the surface of the fresh TMR and were re-fed to the kids. The amount of feed offered was adjusted daily in the morning to ensure a 10% refusal (as fed basis). Samples of TMR were collected at the beginning of each period and stored at − 20 °C for chemical analysis. At the end of the experiment, two kids from each experimental unit (total 8 per treatment) were randomly selected and slaughtered by exsanguination. Before slaughter, the kids were prevented from consuming feed for 24 h and from drinking for 2 h. Immediately after death, the rumen was dissected and digesta was squeezed through two layers of cheesecloth. Left *Longissimus dorsi* and left *Biceps femoris* were collected. Subsamples of rumen liquid (500 mL) and muscle (100 g) were snap frozen in liquid N_2_ and stored at − 80 °C until analysis.

### Chemical analyses

#### Analysis of feed

Samples of dietary ingredients were analyzed for DM (method 930.15), ether extract (method 920.39), CP (N × 6.25; method 984.13), calcium and phosphorous (method 935.13) according to AOAC [13]. Neutral detergent fibre and ADF were determined according to the methods described by Van Soest et al. [14] with an Ankom 220 Fiber Analyser (Ankom Co., USA) and were expressed inclusive of residual ash. Heat stable amylase was not used in the NDF determination.

#### Measurement of FA

Fatty acid methyl esters were produced from samples of feed, plasma, muscle, and rumen liquid, according to the method of O’Fallon et al. [15], and were analyzed as described previously [16].

### RNA extraction and real-time PCR

For *Longissimus dorsi* only, total RNA was extracted from 0.5 g samples of frozen tissue using the RNAiso Reagent (TaKaRa, Dalian, China) according to the manufacturer’s recommendations. The concentration, purity and integrity of the RNA were assessed by 2% agarose gel electrophoresis and a microplate reader (Synergy H4, BioTek, USA) at 260/280 nm (OD260/OD280 = 1.8–2.0). Synthesis of first-strand cDNA and quantitative real-time PCR were performed as described previously [16, 17]), with the same primer pair sequences, and the same three gene references (β-2-microglobulin, tyrosine 3-monooxygenase, β-actin). The primers used are presented in Supplementary Table S1. The efficiency of PCR amplification for each gene was calculated with the standard curve method (E = 10 ^− 1^/slope). The efficiency of PCR amplification for all genes was between 0.98–0.99 (Supplementary Table S1). The 2^−ΔΔCt^ method was used to analyze the qPCR data [18]. The qPCR data were normalized using the geometric mean Ct of the three reference genes [19].

### Metagenomic analyses

#### DNA extraction

Microbial DNA was extracted from rumen samples from six of the eight slaughtered kids in each group, using the E.Z.N.A.® soil DNA Kit (Omega Biotek, Norcross, GA, U.S.) according to the manufacturer’s protocols. DNA integrity was evaluated using 1% agarose gel electrophoresis. The DNA was diluted to 1 ng/μL using sterile water. The extraction and the metagenomic analyses were conducted in Inner Mongolia Agriculture University.

#### Hiseq sequencing and data analysis

Polymerase chain reaction was used to amplify the V4 region of the bacterial 16S rRNA gene using the universal primers 515F (5´-GTGCCAGCMGCCGCGGTAA-3′) and 806R (5´-GGACTACHVGGTWTCTAAT-3′; with the barcode [20]. The forward primer contained 6-base barcode sequences. The reaction was carried out with Phusion® High-Fidelity PCR Master Mix (New England Biolabs). The PCR reaction mixture (30 μL) contained 10 μL DNA template, 15 μL of Phusion Master Mix (2×), 1.5 μL of each primer (total 6 μmol/L and 2 μL of double-distilled H_2_O. The PCR was performed under the following conditions: 98 °C for 1 min, followed by 30 cycles of 98 °C for 10 s, 50 °C for 30 s, and 72 °C for 30 s, and a final elongation step of 72 °C for 5 min. The same volume of 1× loading buffer (contained SYB green) was mixed with the PCR products and the mixture was subjected to electrophoresis on 2% agarose gel. Samples with a bright main strip between 400 and 450 bp were chosen for further analysis. The PCR products were mixed in equimolar amounts and then purified with a Qiagen Gel Extraction Kit (Qiagen, Germany). Sequencing libraries were generated using TruSeq® DNA PCR-Free Sample Preparation Kit (Illumina, USA), following the manufacturer’s recommendations, and index codes were added. Library quality was assessed on the Qubit@ 2.0 Fluorometer (Thermo Scientific) and Agilent Bioanalyzer 2100 system. Finally, the library was sequenced on an Illumina HiSeq 2500 platform and 250 bp paired-end reads were generated.

The generated raw sequences were processed using FLASH and Trimmomatic to merge the paired-end sequences and remove low quality reads with the following criteria: i) The reads were truncated at any site receiving an average quality score < 20 over a 50-bp sliding window; ii) Primer matching allowed 2-nucleotide mismatching, and reads containing ambiguous bases were removed; iii) Sequences with an overlap longer than 10 bp were merged according to their overlap sequence. Operational taxonomic units (OTUs) were clustered with a 97% similarity cut-off using UPARSE (version 7.1 http://drive5.com/uparse/) and chimeric sequences were identified and removed using UCHIME. The taxonomy of each 16S rRNA gene sequence was analyzed with the RDP Classifier algorithm (http://rdp.cme.msu.edu/) against the Silva 128 16S rRNA database (Release128 http://www.arb-silva.de) using a confidence threshold of 70%. Bacterial diversity was measured using the QIIME pipeline based on the OTUs [21]. To eliminate variation among individual kids and thus allow all samples to be compared at the same OTU sequence number, OTU abundance information was normalized using a standard sequence number corresponding to the sample with the least sequences. Subsequent analyses were performed on this output-normalized data.

### Statistical analysis

The data for muscle FA composition were analyzed using PROC MIXED of SAS (version 9.2, SAS Institute Inc., Cary, NC). The MIXED statistical model used for analysis was y_ijkl_ = μ + L_i_ + E_j_ + LE_ij_ + A_ijk_ + T_l_ + TL_il_ + TE_jl_ + TLE_ijl_ + ε_ijkl_ where y_ijkl_ was the dependent, continuous variable, μ is the overall mean, L_i_ was the fixed effect of diet (i = palm oil, linseed oil or mixed oil), E_j_ was the fixed effect of tissue (j = *longissimus dorsi* or *biceps femoris*), A_ijk_ was the random effect of the k^th^ pen in the ij^th^ combination of diet and tissue, T_l_ was the random effect of pen, the two- and three-way interactions of diet, tissue and pen were all considered fixed effects, and ε_ijkl_ was the residual error. Pen was considered as the experimental unit. Tissue was a repeated measurement. Least square means were compared using LSD and statistical differences were declared significant at *P* < 0.05, and tendencies are discussed at 0.05 ≤ *P* < 0.10.

Dry matter intake, rumen FA, plasma FA, and mRNA expression were analyzed using the MIXED procedure. The statistical model included treatment as fixed effects, and pens were added to the model as random effects. Specifically, the model used to study DMI consider 4 replicates (number of pens), each with 1 observation; rumen FA, plasma FA, and mRNA expression consider 4 replicates (number of pens), each with 2 observations (number of goats), for each treatment. The effects of fixed factors were declared significant at *P* < 0.05, and tendencies are discussed at 0.05 ≤ *P* < 0.10.

Multivariate analysis was carried out using R software with the nonparametric MANOVA (Adonis) add-on. Adonis was performed on the Weighted Unifrac distances to assess the significance of differences in bacterial community structure across treatments at a significance level of α = 0.05 based on 9999 possible permutations. Non-metric multidimensional scaling (nMDS) plots were constructed to visualize the data. The ternary plot was created with GGTERN. For indices of bacterial diversity, ANOVA and *post hoc*, Tukey HSD tests were carried out. The results are presented as the mean and standard error of the mean (SEM). Data means were considered significantly different at *P* < 0.05.

The rates of reduction in C18:3n-3 and *cis-*9 C18:1 from dietary values (d 61–90) to rumen values, were considered to reflect hydrogenation [6, 22–24]. The rate of increment in the proportion of C18:0 from diet (d 61–90) to rumen was considered to reflect synthesis. The six rumen samples used for analysis of bacterial community structure in each group were also used to calculate the rates of reduction and increment in FAs. ANOVA and post hoc Tukey HSD tests were carried out. Spearman correlation analysis was used to relate the abundance of the top 45 bacterial genera and the rate of hydrogenation of dietary C18:3n-3 and *cis-*9 C18:1, and the rate of synthesis of C18:0, using R (pheatmap package). Only correlations with *P* < 0.05 for the linear model were considered as significant.

## Results

### Rumen FA composition

Compared with the LSO diet, the PMO or MIX diets increased (*P* < 0.05) the rumen proportion of C16:0, but decreased the proportion of C18:3n-3 (*P* < 0.05). The rumen C16:0 proportion did not differ (*P ≥* 0.10) between the PMO and MIX treatments (Table 2), but the C18:3n-3 value was greater in MIX-fed rumen than in PMO-fed rumen (*P* < 0.05). Rumen proportion of *cis-*9 C18:1 was reduced (*P* < 0.05) in LSO kids compared with PMO kids. The *cis-*9 C18:1 values for MIX kids did not differ (*P ≥* 0.10) from those for either PMO or LSO kids. Rumen proportions of C21:0 and C22:1 were reduced (*P* < 0.05) whereas the proportions of C17:1 (*P =* 0.067), C24:1 (*P =* 0.058), C20:2n-6 (*P =* 0.070) only tended to decrease in MIX kids compared with PMO kids. The proportions of C21:0, C22:1, C17:1, C24:1, and C20:2n-6 for LSO kids did not differ (*P ≥* 0.10) from those for either PMO or MIX kids. The proportion of C18:2n-6*c* tended to increase in MIX kids compared with PMO kids (*P =* 0.098), but the value for LSO kids did not differ (*P ≥* 0.10) from those for either PMO or MIX kids.

**Table 2:** Fatty acid profiles (percentage of total identified fatty acids methyl esters) in rumen of kids fed diets containing oil supplements

Fatty acid	PMO	LSO	MIX	SEM	*P*-value
Saturated fatty acids					
C10:0	1.57	1.58	1.42	0.107	0.526
C12:0	2.02	2.20	2.04	0.09	0.408
C13:0	0.96	1.02	0.87	0.049	0.150
C14:0	3.99	3.74	3.59	0.162	0.331
C15:0	2.03	2.15	2.22	0.095	0.465
C16:0	15.27^a^	13.65^b^	14.81^a^	0.171	<.001
C17:0	2.00	1.87	1.92	0.072	0.451
C18:0	16.72	18.21	18.86	0.535	0.103
C20:0	2.53	2.36	2.4	0.06	0.346
C21:0	0.90^a^	0.86^ab^	0.72^b^	0.042	0.046
C22:0	2.26	1.93	1.96	0.129	0.205
C23:0	0.82	0.85	0.70	0.049	0.142
C24:0	2.04	1.97	1.89	0.075	0.436
Monounsaturated fatty acids					
C14:1	1.62	1.62	1.66	0.061	0.896
C15:1	0.73	0.74	0.79	0.043	0.556
C16:1	1.98	1.79	1.98	0.103	0.399
C17:1	1.14	0.90	0.75	0.094	0.067
*tran*-9 18:1	8.82	10.69	9.94	0.469	0.149
*cis*-9 C18:1	11.44^a^	9.35^b^	10.45^ab^	0.342	0.009
C20:1	1.12	0.94	0.97	0.079	0.295
C22:1	0.90^a^	0.87^ab^	0.71^b^	0.048	0.040
C24:1	0.96	0.89	0.76	0.046	0.058
n-6 Polyunsaturated fatty acids					
C18:2n-6*t*	0.88	0.83	0.75	0.034	0.151
C18:2n-6*c*	4.76	5.65	6.14	0.306	0.098
C18:3n-6	0.87	0.85	0.83	0.039	0.808
C20:2n-6	0.88	0.82	0.71	0.041	0.070
C20:3n-6	0.79	0.8	0.73	0.042	0.444
C20:4n-6	0.85	0.86	0.79	0.042	0.469
C22:2n-6	0.88	0.84	0.69	0.046	0.045
n-3 Polyunsaturated fatty acids					
C18:3n-3	1.36^c^	2.15^a^	1.71^b^	0.050	<.001
C20:3n-3	0.79	0.81	0.71	0.053	0.720
C20:5n-3	0.83	0.82	0.70	0.048	0.200
C22:6n-3	0.86	0.89	0.76	0.056	0.302
Total fat (% w/w of rumen liquid)	0.015	0.016	0.014	0.001	0.582
Proportional loss rate of dietary fatty acids, %					
*cis-*9 C18:1	63.14^a^	35.74^c^	47.85^b^	1.04	<.001
C18:3n-3	76.42^c^	94.65^a^	92.96^b^	0.33	<.001
Proportional increase rate of dietary fatty acids, %					
C18:0	576.85^a^	488.92^b^	559.63^a^	18.29	0.009
Dry matter intake, kg per day per pen	4.15	4.30	3.70	0.35	0.478

*PMO* palm oil diet, *LSO* linseed oil diet, *MIX* mixed oil diet

^a–c^ Means within the same row followed by the same superscript letters are not significantly different at *P* < 0.05

### Muscle FA composition

There were significant interactions (*P* < 0.05) between oil type and muscle for concentrations of C21:0, C17:1, C18:2n-6*t*, C18:3n-6, C20:2n-6, C18:3n-3, C22:6n-3, and the n-6/n-3 ratio (Table 3). For all dietary treatments, the values for C18:2n-6*t* concentration and n-6/n-3 ratio were significantly greater (*P* < 0.05) in *Biceps femoris* than in *Longissimus dorsi.* Values for C18:3n-6 concentration and C20:2n-6 in *Biceps femoris* were significantly lower (*P* < 0.05) with MIX than with the other treatments. The highest C21:0 concentration was observed in *Biceps femoris* with LSO treatment. The lowest C17:1 concentration was observed in *Longissimus dorsi* with PMO treatment. The lowest C18:3n-3 concentrations were observed in both muscles with PMO treatment, while the highest C18:3n-3 concentrations were observed in *Biceps femoris* with MIX treatment, and in *Biceps femoris* with LSO treatment. The concentration of C22:6n-3 was significantly higher in *Longissimus dorsi* with LSO treatment, and in *Longissimus dorsi* with MIX treatment (*P* < 0.05).

**Table 3:** Fatty acid profiles (percentage of total identified fatty acids methyl esters) in muscles of kids fed diets containing oil supplements

	*Longissimus dorsi*			*Biceps femoris*			SEM main effects	*P*-value		
	PMO	LSO	MIX	PMO	LSO	MIX		Diet	Tissue	Diet×Tissue
Total fat content (g/100 g fresh sample)	2.92	3.05	2.78	2.57	2.55	2.53	0.23	0.817	0.066	0.884
Saturated fatty acids										
C10:0	0.76	0.87	0.75	1.46	1.49	1.61	0.114	0.601	<.0001	0.212
C12:0	0.72	0.88	0.71	1.38	1.49	1.36	0.129	0.061	<.0001	0.892
C13:0	0.51	0.56	0.55	0.83	0.76	0.89	0.080	0.953	<.0001	0.663
C14:0	1.75	2.01	2.10	2.40	2.79	2.51	0.261	0.055	<.0001	0.379
C15:0	0.57	0.67	0.59	0.76	0.86	0.76	0.066	0.011	<.0001	0.896
C16:0	17.2	16.72	17.54	17.05	17.06	17.1	1.041	0.701	0.889	0.768
C17:0	4.89	5.16	5.24	1.36	1.49	1.42	0.428	0.534	<.0001	0.808
C18:0	12.9	12.54	12.50	11.91	11.87	12.00	0.824	0.855	0.036	0.846
C20:0	1.32	1.36	1.29	1.33	1.50	1.28	0.169	0.241	0.440	0.641
C21:0	0.96^c^	1.04^bc^	0.89^c^	0.97^c^	1.18^a^	1.09^ab^	0.072	0.001	<.0001	0.049
C22:0	0.91	1.05	0.91	1.37	1.48	1.36	0.168	0.152	<.0001	0.974
Monounsaturated fatty acids										
C14:1	0.41	0.47	0.48	0.67	0.72	0.65	0.076	0.268	<.0001	0.398
C15:1	0.45	0.51	0.54	0.66	0.71	0.63	0.087	0.456	<.0001	0.377
C16:1	1.63	1.73	1.85	2.15	2.03	2.25	0.141	0.040	<.0001	0.316
C17:1	0.99^b^	1.07^ab^	1.25^a^	1.16^ab^	1.22^a^	1.11^ab^	0.141	0.274	0.218	0.047
*tran-*9 C18:1	0.99	1.00	1.05	0.99	1.03	1.01	0.098	0.779	0.905	0.905
*cis-*9 C18:1	39.05	36.82	36.44	33.10	32.08	32.74	1.664	0.132	<.0001	0.332
C20:1	0.58	0.65	0.52	0.73	0.77	0.64	0.074	0.005	<.0001	0.963
C24:1	0.50	0.59	0.52	0.82	0.80	0.82	0.125	0.904	<.0001	0.639
n-6 Polyunsaturated fatty acids										
C18:2n-6*c*	0.54	0.64	0.51	0.59	0.64	0.60	0.075	0.047	0.150	0.585
C18:2n-6*t*	3.61^c^	3.64^c^	3.42^c^	7.09^a^	6.04^b^	6.69^a^	0.423	0.340	<.0001	0.007
C18:3n-6	0.56^a^	0.60^a^	0.60^a^	0.57^a^	0.61^a^	0.46^b^	0.051	0.022	0.061	0.008
C20:2n-6	0.68^ab^	0.76^a^	0.73^ab^	0.65^b^	0.68^ab^	0.50^c^	0.072	0.023	0.001	0.041
C20:3n-6	0.77	0.78	0.71	0.82	0.78	0.72	0.087	0.119	0.700	0.834
C20:4n-6	2.22	2.29	2.14	4.41	3.56	3.94	0.472	0.312	<.0001	0.167
C22:2n-6	0.72	0.95	0.81	0.61	0.77	0.76	0.097	0.011	0.031	0.302
n-3 Polyunsaturated fatty acids										
C18:3n-3	1.39^d^	1.52^cd^	1.71^c^	1.35^d^	2.47^a^	2.10^b^	0.135	<.0001	<.0001	<.0001
C20:3n-3	0.79	0.91	0.97	0.63	0.67	0.59	0.085	0.150	<.0001	0.058
C20:5n-3	1.12	1.22	1.51	1.39	1.62	1.62	0.154	0.002	0.001	0.256
C22:6n-3	0.86^c^	1.04^b^	1.25^a^	0.77^c^	0.76^c^	0.86^c^	0.096	0.001	<.0001	0.015
Sum and ratio										
SFA	42.47	42.82	43.01	40.85	42.00	41.34	0.900	0.253	0.001	0.570
MUFA	44.66	42.83	42.65	40.28	39.4	39.85	1.199	0.050	<.0001	0.393
PUFA	12.88	14.35	14.34	18.87	18.61	18.81	1.254	0.492	<.0001	0.336
n-3 PUFA	4.17	4.69	5.44	4.14	5.52	5.17	0.624	0.003	0.495	0.206
n-6 PUFA	8.71	9.65	8.91	14.73	13.09	13.64	1.079	0.771	<.0001	0.077
n-3 LCPUFA	2.78	3.16	3.73	2.78	3.05	3.07	0.537	0.089	0.246	0.432
n-6 LCPUFA	4.41	4.79	4.38	6.48	5.78	5.91	0.569	0.587	<.0001	0.189
n-6/n-3	2.09^d^	2.06^d^	1.64^e^	3.55^a^	2.37^c^	2.64^b^	0.131	<.0001	<.0001	<.0001
U/S	1.35	1.33	1.33	1.45	1.38	1.42	0.079	0.576	0.019	0.702
P/S	0.30	0.34	0.33	0.46	0.44	0.46	0.040	0.754	<.0001	0.309

^a,b,c,d,e^ Interactions of diet and tissue means within a row without a common superscript letter differ (*P* < 0.05)

^1^ SFA saturated fatty acids (10:0 + 11:0 + 12:0 + 13:0 + 14:0 + 15:0 + 16:0 + 17:0 + 18:0 + 20:0 + 21:0 + 22:0 + 23:0 + 24:0), MUFA monounsaturated fatty acids (14:1 + 15:1 + 16:1 + 17:1 + *tran*-9 18:1 + *cis*-9 18:1 + 20:1 + 22:1 + 24:1), n-6 PUFA n-6 polyunsaturated fatty acids (18:2n-6*t* + 18:2n-6*c* + 18:3n-6 + 20:2n-6 + 20:3n-6 + 20:4n-6 + 22:2n-6), n-3 PUFA n-3 polyunsaturated fatty acids (18:3n-3 + 20:3n-3 + 20:5n-3 + 22:6n-3), n-6 LCPUFA n-6 long chain polyunsaturated fatty acids (20:2n-6 + 20:3n-6 + 20:4n-6 + 22:2n-6), n-3 LCPUFA n-3 long chain polyunsaturated fatty acids (20:3n-3 + 20:5n-3 + 22:6n-3); n-6/n-3 n-6 long chain polyunsaturated fatty acids/n-3 long chain polyunsaturated fatty acids; P/S polyunsaturated fatty acids/saturated fatty acids; U/S unsaturated fatty acids/saturated fatty acids

*PMO* palm oil diet, *LSO* linseed oil diet, *MIX* mixed oil diet

**Table 4:** Fatty acid profiles (percentage of total identified fatty acid methyl esters) in plasma of cashmere goat kids fed diets with oil supplements

Fatty acid^1^	Diets			SEM	*P*-value
	PMO	LSO	MIX		
Saturated fatty acids					
C10:0	1.47	1.82	1.43	0.124	0.352
C12:0	1.49	1.68	1.45	0.093	0.383
C13:0	0.63	0.78	0.69	0.056	0.438
C14:0	2.20	2.26	2.03	0.090	0.401
C15:0	1.27	1.21	1.22	0.094	0.900
C16:0	15.85^a^	13.00^b^	14.31^ab^	0.508	0.008
C17:0	1.94	1.83	1.89	0.079	0.714
C18:0	17.10	18.46	18.90	0.800	0.518
C20:0	1.62	1.80	1.46	0.067	0.133
C21:0	0.82^b^	1.06^a^	0.96^ab^	0.038	0.018
C22:0	1.75	1.80	1.62	0.091	0.545
Monounsaturated fatty acids					
C14:1	1.04	1.07	0.93	0.073	0.652
C15:1	0.69	0.95	0.71	0.057	0.081
C16:1	1.69^a^	1.45^b^	1.61^ab^	0.047	0.063
C17:1	1.34	1.28	1.32	0.091	0.954
*tran-*9 C18:1	1.62	1.60	1.49	0.089	0.772
*cis-*9 C18:1	22.47^a^	17.91^b^	19.92^ab^	0.901	0.035
C20:1n-9	0.74^b^	1.01^a^	0.71^b^	0.046	0.012
C22:1n-9	0.56	0.88	0.76	0.068	0.154
n-6 Polyunsaturated fatty acids					
C18:2n-6*t*	0.95	1.06	1.10	0.051	0.514
C18:2n-6*c*	11.46	11.27	10.97	0.380	0.809
C18:3n-6	1.00	1.10	0.91	0.052	0.195
C20:2n-6	0.83	1.01	0.83	0.048	0.113
C20:3n-6	1.07	0.92	0.94	0.034	0.758
C20:4n-6	1.79	1.99	1.97	0.057	0.689
C22:2n-6	0.81	0.99	0.80	0.045	0.168
n-3 Polyunsaturated fatty acids					
C18:3n-3	2.51^b^	3.80^a^	3.63^a^	0.130	0.001
C20:3n-3	0.53	0.83	0.72	0.046	0.125
C20:5n-3	1.01^b^	2.13^a^	2.04^a^	0.046	0.0001
C22:6n-3	0.87^b^	1.28^a^	1.32^a^	0.066	0.020
Sum and ratio					
SFA	46.72	46.68	46.86	0.743	0.987
MUFA	30.84^a^	26.97^b^	28.23^ab^	0.949	0.081
PUFA	22.45^b^	26.38^a^	25.00^ab^	0.539	0.007
n-3 PUFA	4.91^b^	8.04^a^	7.71^a^	0.249	<.0001
n-6 PUFA	17.53	18.34	17.19	0.567	0.490
n-3 LCPUFA	2.40^b^	4.25^a^	4.09^a^	0.196	<.0001
n-6 LCPUFA	4.49	4.92	4.54	0.290	0.344
n-6/n-3	3.57^a^	2.28^b^	2.23^b^	0.120	<.0001
P/S	0.49	0.58	0.53	0.026	0.227

^a,b^ Means within the same row followed by the same superscript are not significantly different at *P* < 0.05

^1^ SFA saturated fatty acids (10:0 + 11:0 + 12:0 + 13:0 + 14:0 + 15:0 + 16:0 + 17:0 + 18:0 + 20:0 + 21:0 + 22:0 + 23:0 + 24:0), MUFA monounsaturated fatty acids (14:1 + 15:1 + 16:1 + 17:1 + *tran-*9 18:1 + *cis-*9 18:1 + 20:1 + 22:1 + 24:1), n-6 PUFA n-6 polyunsaturated fatty acids (18:2n-6*t* + 18:2n-6*c* + 18:3n-6 + 20:2n-6 + 20:3n-6 + 20:4n-6 + 22:2n-6), n-3 PUFA n-3 polyunsaturated fatty acids (18:3n-3 + 20:3n-3 + 20:5n-3 + 22:6n-3), n-6 LCPUFA n-6 long chain polyunsaturated fatty acids (20:2n-6 + 20:3n-6 + 20:4n-6 + 22:2n-6), n-3 LCPUFA n-3 long chain polyunsaturated fatty acids (20:3n-3 + 20:5n-3 + 22:6n-3); n-6/n-3 n-6 long chain polyunsaturated fatty acids/n-3 long chain polyunsaturated fatty acids; P/S polyunsaturated fatty acids/saturated fatty acids

*PMO* palm oil diet, *LSO* linseed oil diet, *MIX* mixed oil diet

Significant main effects are also shown in Table 3. Compared with *Biceps femoris*, total fat content tended to be greater in *Longissimus dorsi* (*P* = 0.066). Dietary supplementation with LSO or MIX increased the muscle concentrations of C18:3n-3 and n-3 PUFA (*P* < 0.05), but decreased the muscle concentration of C16:1 (*P* < 0.05) and the n-6/n-3 ratio (*P* < 0.05), and tended to decrease the muscle concentration of MUFA (*P* = 0.066), compared with PMO. The muscle concentrations of those FAs did not differ (*P ≥* 0.10) between kids fed the LSO and MIX diets.

Muscle concentrations of C20:5n-3 were greater (*P* < 0.05) and concentrations of n-3 LCPUFA tended to be greater (*P* = 0.098), in MIX kids compared with PMO kids, but the value for LSO kids did not differ from those for either PMO or MIX kids (*P ≥* 0.10). Diets containing PMO or LSO alone reduced the concentration of C22:6n-3 compared with the MIX diet (*P* < 0.05). Muscle concentration of C22:6n-3 did not differ between kids fed LSO or PMO alone (*P ≥* 0.10). Muscle concentrations of C15:0, C18:2n-6*t*, C21:0, and C20:1 were greater in LSO-fed kids compared with PMO and MIX-fed kids (*P* < 0.05), but did not differ (*P ≥* 0.10) between PMO and MIX kids. Muscle concentrations of C18:3n-6 and C20:2n-6 were lower in MIX-fed kids than in LSO-fed kids (*P* < 0.05). The C18:3n-6 and C20:2n-6 values for PMO kids did not differ (*P ≥* 0.10) from those for either LSO or MIX kids. Compared with PMO-fed kids, muscle concentration of C22:2n-6 were lower in LSO-fed kids (*P* < 0.05), but the value for MIX kids did not differ from those for either PMO kids or LSO kids (*P ≥* 0.10).

As shown in Table 3, compared with *Biceps femoris*, *Longissimus dorsi* concentrations of C17:0, C18:0, *cis-*9 C18:1, C20:2n-6, C22:2n-6, saturated fatty acids (SFA) and monounsaturated fatty acids (MUFA) were greater (*P* < 0.05), and the concentration of C18:3n-6 tended to be greater (*P* = 0.061), whereas the concentrations of C10:0, C12:0, C13:0, C14:0, C21:0, C22:0, C14:1, C15:1, C16:1, C20:1, C24:1, C18:2n-6*c*, C20:4n-6, C18:3n-3, C20:5n-3, PUFA, n-6 PUFA, and n-6 LCPUFA were lower, as were the ratios for n-6/n-3, unsaturated fatty acid/saturated fatty acid (U/S), and polyunsaturated fatty acid/saturated fatty acid (P/S; *P* < 0.05).

### Plasma FA composition

Compared with the LSO diet, supplementation with PMO increased (*P* < 0.05) the plasma proportions of C16:0 and *cis*-9 18:1, and tended to increase the proportions of C16:1 (*P =* 0.063) and MUFA (*P =* 0.081), but decreased (*P* < 0.05) the plasma proportions of C21:0 and PUFA (Table 4). Plasma proportions of C18:3n-3, C20:5n-3, C22:6n-3, n-3 PUFA, n-3 LCPUFA were reduced (*P* < 0.05), but the n-6/n-3 ratio was increased (*P* < 0.05) in PMO kids compared with LSO and MIX kids. The values of C18:3n-3, C20:5n-3, C22:6n-3, n-3 PUFA, n-3 LCPUFA, and the n-6/n-3 ratio did not differ (*P ≥* 0.10) between kids fed the LSO and MIX diets. Plasma proportion of C20:1n-9 was increased (*P* < 0.05) in LSO kids compared with PMO and MIX kids. The value of C20:1n-9 did not differ (*P ≥* 0.10) between kids fed the PMO and MIX diets.

### mRNA expression

**Fig. 1:**
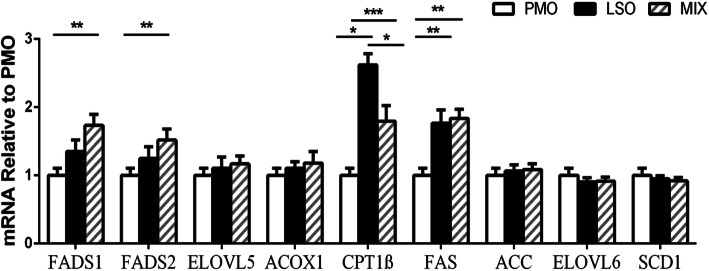
Relative expression of genes related to lipid metabolism in muscle (*Longissimus dorsi*) of cashmere goat kids fed diets with oil supplements. PMO, palm oil diet; LSO, linseed oil diet; MIX, mixed oil diet. *FAS* = fatty acid synthetase, *ACC* = acetyl-CoA carboxylase, *SCD1* = stearoyl-CoA desaturase 1, *FADS1* = delta-5 desaturase, *FADS2* = delta-6 desaturase, *ELVOL5* = elongation of very long chain fatty acids protein 5, *ELOVL6* = elongation of very long chain fatty acids protein 6, *ACOX1* = acyl-coenzyme A oxidase 1, *CPT1β* = carnitine palmitoyltransferase I. ^*^P < 0.05, ^**^P < 0.01, ^***^P < 0.001

The relative mRNA expression of genes in *Longissimus dorsi* is presented in Fig. 1. In comparison to PMO, mRNA expression of fatty acid synthetase (*FAS*) was greater (*P* < 0.05) with both the LSO and MIX treatments, with no difference (*P ≥* 0.10) between MIX and LSO kids. In comparison to the LSO treatment, mRNA expression of carnitine palmitoyltransferase I (*CPT1β*) was lower with the PMO and MIX treatments (*P* < 0.05), and mRNA expression of *CPT1β* was greater with the MIX treatment than with the PMO treatment (*P* < 0.05). Kids fed the mixed oil diet showed greater mRNA expression of *FADS1* and *FADS2* than PMO-fed kids, but LSO-fed kids did not differ (*P ≥* 0.10) from either PMO-fed or MIX-fed kids.

### Bacterial diversity

#### Sequencing coverage and bacterial diversity

A total of 1,336,287 reads were generated after quality control and chimera removal, resulting in an average of 74,238 reads per sample, with sequence numbers per sample ranging from 51,620 to 85,348 (median 74,632). A total of 418 unique OTUs that could be taxonomically classified to genus level were identified across all samples. The OTU rarefaction curves of the bacterial communities in the ruminal digesta show that the sampling effort was sufficient to estimate bacterial diversity (Supplementary Fig. S1). Alpha diversity indices (Table 5) indicated that supplementation did not significantly affect OTU number, ACE, Chao, Simpson, Shannon, and coverage indices (*P ≥* 0.10).

**Table 5:** Alpha diversity indices of ruminal bacteria in kids fed different diets

Index	PMO	LSO	MIX	SEM	*P*-value
Observed OTUs	1739.5	1790.83	1790.33	35.57	0.518
Shannon	5.45	5.43	5.39	0.13	0.952
Simpson	0.02	0.02	0.02	0.01	0.658
ACE	2159.59	2212.18	2245.91	34.58	0.238
Chao	2172.17	2229.2	2267.18	40.01	0.270
Good’s coverage	0.99	0.99	0.99	0.0002	0.123

*PMO* palm oil diet, *LSO* linseed oil diet, *MIX* mixed oil diet

#### Bacterial community

**Fig. 2:**
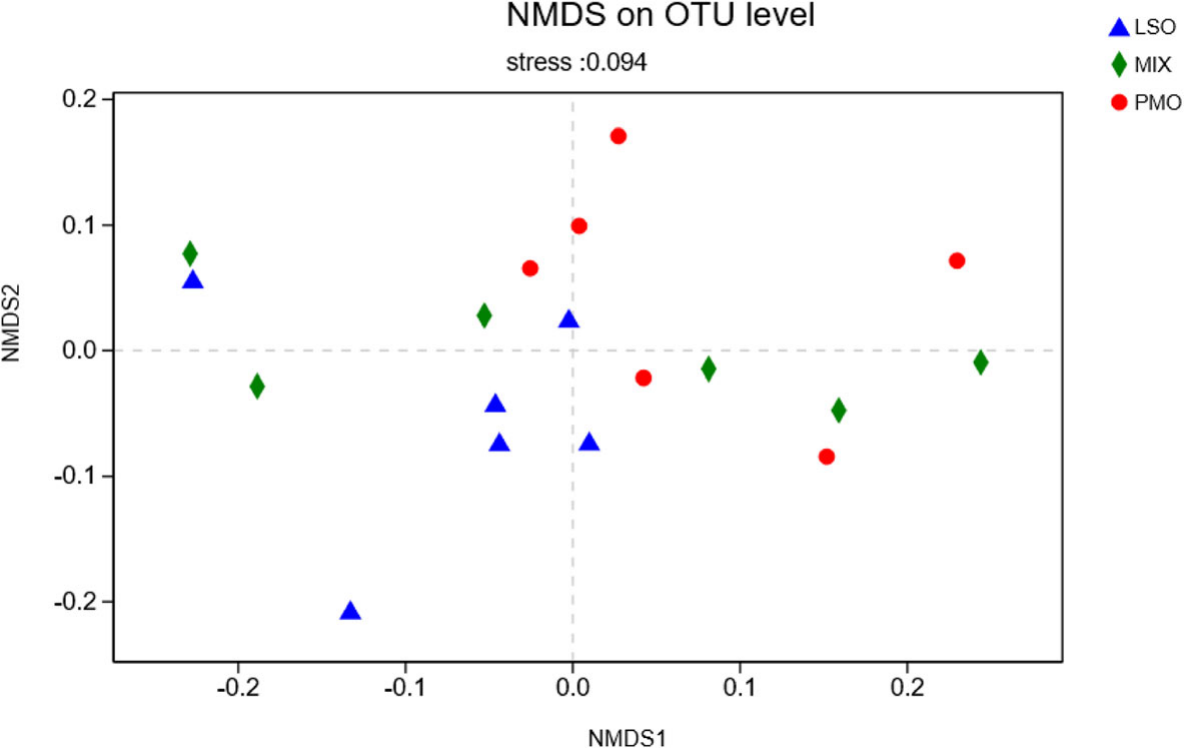
Non-metric multidimensional scaling (nMDS) plot of rumen bacterial community structures. Each color represents a dietary treatment: PMO (red); LSO (blue); MIX (green)

As reflected by nMDS using the weighted Unifrac similarity metric, the samples clustered according to the dietary treatments (Fig. 2). There is a clear separation between PMO and LSO (Adonis analysis, *P* < 0.001), whereas the points for the MIX animals were spread.

#### Spearman correlation analysis

**Fig. 3:**
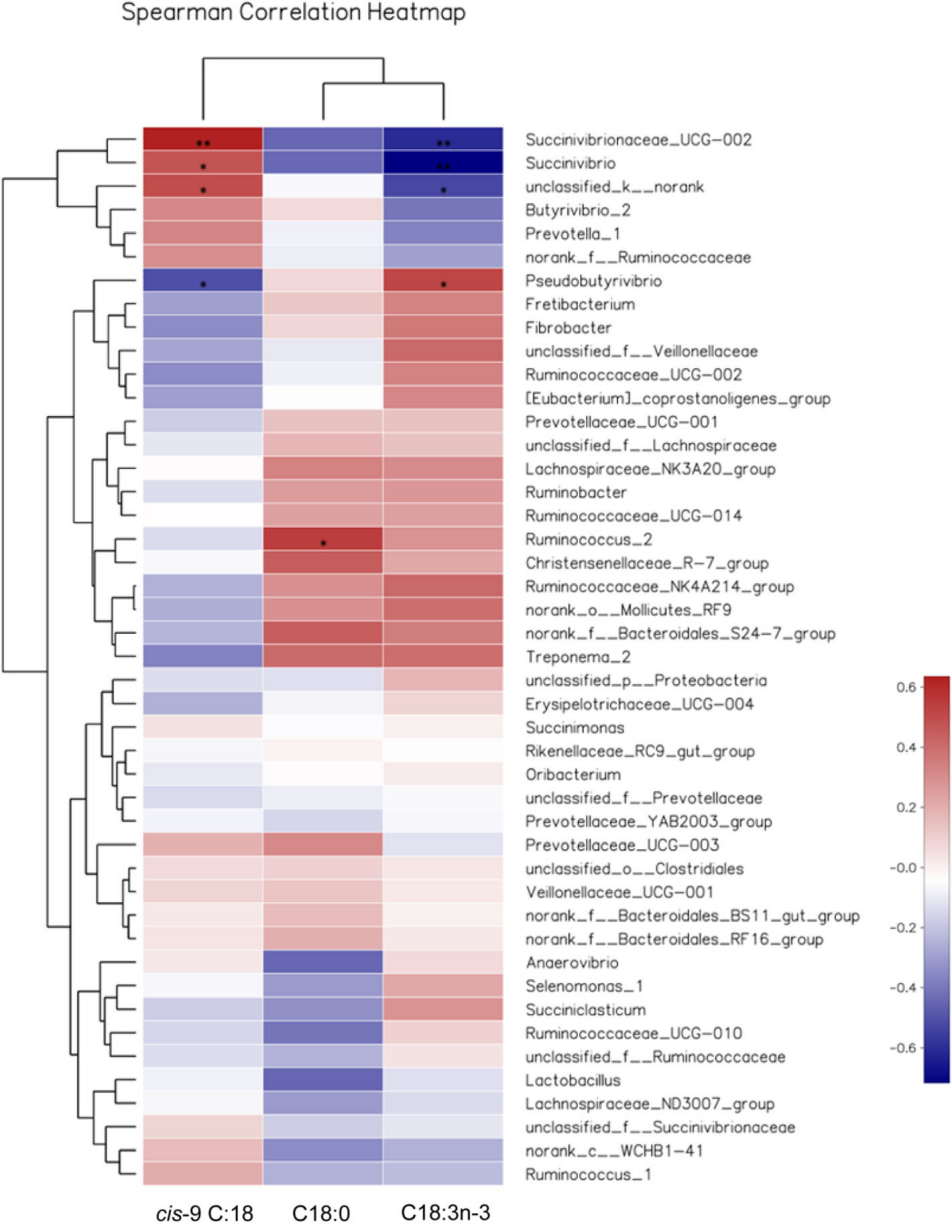
Heat maps showing correlations between the relative abundance of sequences assigned to each bacterial genus and hydrogenation rate of fatty acids (C18:3n-3 and *cis-*9 C18:1) in the rumen of kids fed palm oil, linseed oil and mixed oil. Spearman correlation coefficients (*r*) are shown in different colors: *r* < 0 (blue), *r* = 0 (white) and *r* > 0 (red). * correlation significant at 0.01 < *P* ≤ 0.05; ** correlation significant at 0.001 < *P* ≤ 0.01

The 45 most abundant genera represent 91% (PMO), 89% (LSO), and 92% (MIX) of the total microbiome. Spearman correlation analysis was conducted between the abundance of the top 45 genera and the proportional loss rate of fatty acids (C18:3n-3 and *cis-*9 C18:1), or the proportional increment rate of fatty acid (C18:0) from diet to rumen (Fig. 3). The threshold |R| > 0.4 is considered as a significant Spearman correlation. Among the top 45 genera, an unclassified genus was clustered among the genera belonging to the Bacteroidetes phylum, and was labelled ‘unclassified_k_norank’ by the phylogenetic analysis software (Supplementary Fig. S2). The results indicated that relative abundance of the *unclassified genera in Bacteroidetes*, *Succinivibrio*, and *Succinivibrionaceae UCG-002* were positively correlated with the rate of reduction in the proportion of *cis-*9 C18:1 from diet to rumen, but negatively correlated with the rate of reduction in the proportion of C18:3n-3 from diet to rumen. The relative abundance of *Pseudobutyrivibrio* was positively correlated with the rate of reduction in the proportion of C18:3n-3, but negatively correlated with the rate of reduction in the proportion of *cis-*9 C18:1. For the relative abundance of *Ruminococcus_2*, there was a positive correlation only with the rate of increase in the proportion of C18:0 from diet to rumen.

#### Microbial composition analysis

Collectively, 26 bacterial phyla, 183 families, 418 genera, and 789 species were identified in the rumen samples. Independent of diets, Bacteroidetes, Firmicutes, Proteobacteria, and Verrucomicrobia were the dominant phyla (Supplementary Fig. S3), comprising about 89% of average relative abundance. The most abundant 20 families are shown in Table 6, representing 92% (PMO), 88% (LSO), and 94% (MIX) of the total microbiome. The relative abundance of Bacteroidales_BS11_gut_group was greater in PMO-fed kids than in LSO-fed and MIX-fed kids (*P* < 0.05), but did not differ (*P ≥* 0.10) between LSO-fed and MIX-fed kids. The relative abundance of Veillonellaceae and Acidaminococcaceae was greater in LSO-fed kids than in PMO-fed and MIX-fed kids (*P* < 0.05), but did not differ between PMO-fed and MIX-fed kids (*P ≥* 0.10). Compared with PMO-fed kids, the relative abundance of Bacteroidales_S24–7_group was greater in MIX-fed kids (*P* < 0.05), but values for LSO-fed kids did not differ (*P ≥* 0.10) from those in either the PMO-fed or MIX-fed kids.

**Table 6:** Effects of dietary oil supplements on relative abundances of the 20 most abundant bacteria (family level) in the rumen of cashmere goat kids

Phylum	Family	PMO	LSO	MIX	SEM	***P***-value
Bacteroidetes	Prevotellaceae	0.31	0.25	0.30	0.033	0.347
	Rikenellaceae	0.06	0.06	0.07	0.008	0.599
	Bacteroidales_BS11_gut_group	0.08^a^	0.05^b^	0.04^b^	0.009	0.013
	Bacteroidales_S24-7_group	0.02^b^	0.03^ab^	0.04^a^	0.004	0.031
	Bacteroidales_RF16_group	0.02	0.02	0.02	0.002	0.519
	Unclassified_f_Bacteroidetes	0.03	0.02	0.03	0.006	0.559
Firmicute	Ruminococcaceae	0.09	0.09	0.10	0.008	0.739
	Veillonellaceae	0.04^b^	0.07^a^	0.04^b^	0.009	<.001
	Lachnospiraceae	0.05	0.06	0.05	0.006	0.812
	Acidaminococcaceae	0.009^b^	0.023^a^	0.007^b^	0.003	0.012
	Lactobacillaceae	0.003	0.002	0.001	0.001	0.420
	Erysipelotrichaceae	0.01	0.01	0.01	0.001	0.530
	Christensenellaceae	0.01	0.01	0.01	0.001	0.723
Proteobacteria	Succinivibrionaceae	0.11	0.12	0.15	0.037	0.683
	unclassified_p_Proteobacteria	0.002	0.003	0.002	0.0004	0.362
Verrucomicrobia	norank_c_WCHB1–41	0.03	0.02	0.03	0.004	0.420
Synergistetes	Synergistaceae	0.01	0.02	0.01	0.002	0.342
Fibrobacteres	Fibrobacteraceae	0.01	0.01	0.01	0.001	0.775
Spirochaetae	Spirochaetaceae	0.01	0.01	0.01	0.001	0.149
Tenericutes	norank_o_Mollicutes_RF9	0.01	0.01	0.01	0.001	0.357

*PMO* palm oil diet, *LSO* linseed oil diet, *MIX* mixed oil diet

^a,b^ Means within the same row followed by the same superscript letters are not significantly different at *P* < 0.05 (*n* = 6 for each mean)

**Fig. 4:**
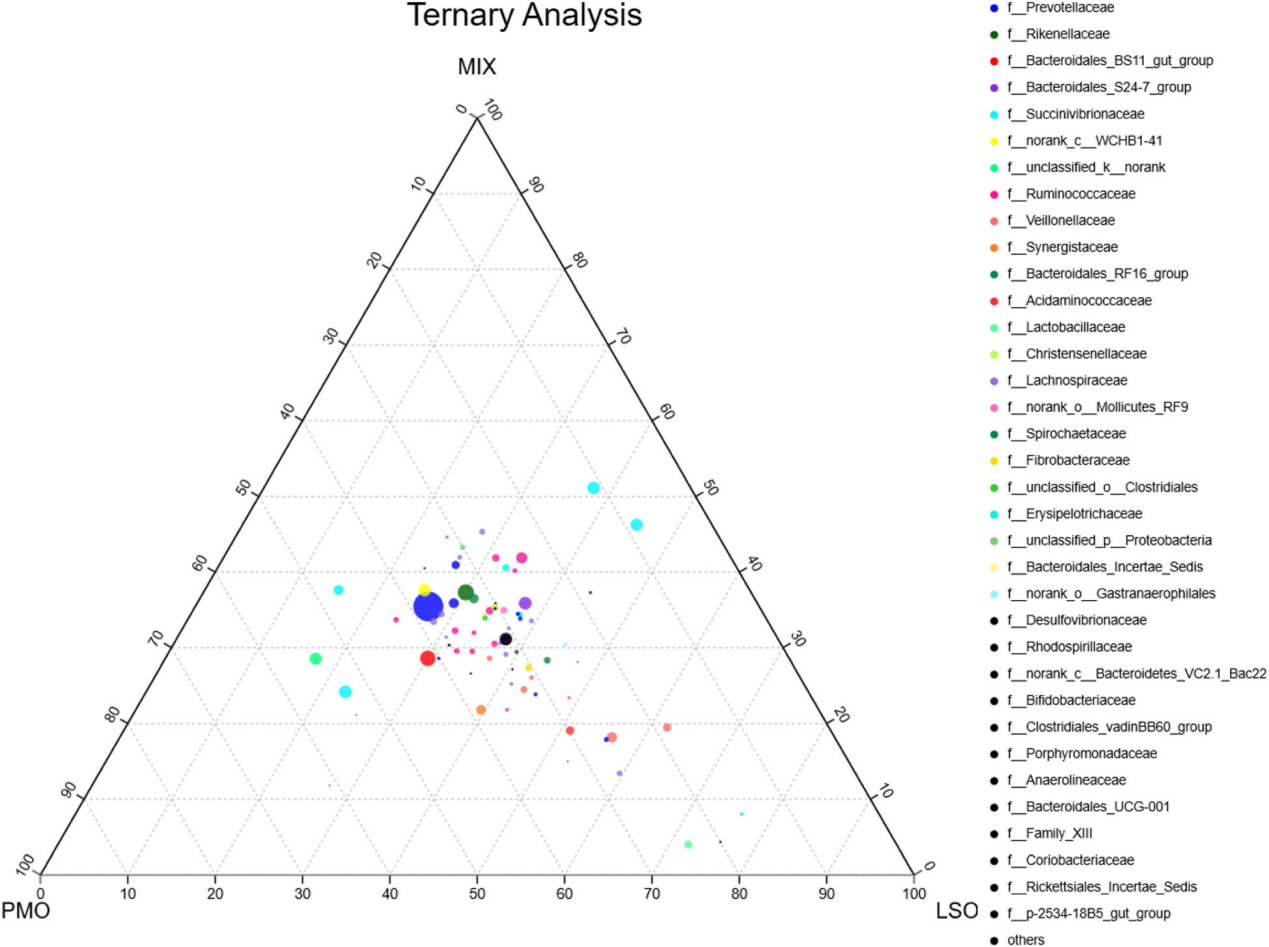
Ternary plot of genus showing the percent of observations for each genus (>0.1%) present in each dietary group (PMO, LSO, MIX). The taxonomic list at family level corresponds to genus of points. For example, a point positioned within the ‘70’ triangle at the ‘LSO’ corner indicates that 70% of all observations of that genus occur within the LSO group. The diameter of plotted points corresponds to relative abundance of the genus. Each compartment of the dotted grid corresponds to a 10% increment

The centre of the ternary plot (Fig. 4) shows the core microbiome (high density of circles) across the PMO, LSO, and MIX treatments. Ternary plot analysis focuses on the abundance of genera that show a Spearman correlation with the proportional changes of fatty acid: the rank from low to high for the total abundance of *Pseudobutyrivibrio* was 27.7% for PMO, 33% for MIX, and 39.3% for LSO, respectively; for *Ruminococcus_2*, the values were 26.9% for PMO, 30.9% for LSO, and 42.2% for MIX, respectively; for *Succinivibrionaceae UCG-002*, the values were 23% for LSO, 23.8% for MIX, and 53.1% for PMO, respectively; for *unclassified genus in Bacteroidetes*, the values were 17% for LSO, 28.5% for MIX, and 54.5% for PMO, respectively; for *Succinivibrio*, the values were 15.3% for LSO, 37.6% for MIX, and 47.1% for PMO, respectively.

## Discussion

The LSO diet contained more C18:3n-3 than the MIX diet, but only the MIX diet increased muscle n-3 LCPUFA concentration to levels greater than those observed with PMO diet. These results are consistent with the literature for goats [3, 25] showing that the C22:6n-3 content of muscle and fat increased as dietary C18:3n-3 content increased with inclusion of palm kernel cake in the diet. For cattle, two observations are relevant: i) the muscle concentration of C22:6n-3 was increased in *Longissimus dorsi* by feeding a diet containing both palm oil and linseed oil, but not diets containing only palm oil diet or linseed oil [26]; ii) a supplement of extruded linseed oil increased the muscle contents of C18:3n-3, but not C22:6n-3 [4]. Our observations support the hypothesis that these outcomes are the result of two processes, one in the rumen and the other in muscle tissue.

### Mixed oil decreases the hydrogenation of dietary C18:3n-3 in rumen

In agreement with a previous study [6], the MIX diet decreased the proportional loss rate of C18:3n-3 but increased the proportional loss rate of *cis-*9 C18:1 compared with LSO diet, leading to an increased post-ruminal C18:3n-3 flow, an expectation that is consistent with the similar plasma concentrations of C18:3n-3 in LSO and MIX kids.The proportional loss rate of FA reflects FA hydrogenation in the rumen [6, 22–24], evidenced by the decreased C18:3n-3 hydrogenation but increased *cis-*9 C18:1 hydrogenation in MIX diet compared with LSO diet. The relative abundance of *Pseudobutyrivibrio* was positively correlated with the rate of reduction in the proportion of dietary C18:3n-3, while the relative abundances of *unclassified genus in Bacteroidetes*, *Succinivibrionaceae UCG-002*, and *Succinivibrio* were positively correlated with the rate of reduction in the proportion of *cis*9 C18:1. Except for *Pseudobutyrivibrio* [27], there is no direct evidence that *unclassified genus in Bacteroidetes, Succinivibrionaceae UCG-002*, and *Succinivibrio* are involved in FA hydrogenation, but these bacteria are thought to provide the energy or hydrogen needed for FA hydrogenation [28–35]. Further studies are needed to confirm the function of FA hydrogentaion of these bacteria.

At family level, Acidaminococcaceae is also involved in the hydrogenation of C18:3n-3 [36]. Veillonellaceae become the most important ruminal bacteria in goats fed a diet supplemented with linseed oil [37], suggesting that they also play an important role in the hydrogenation of dietary C18:3n-3, perhaps because they can provide hydrogen and energy [38].

Ternary plot analysis showed that MIX-fed kids had a lower abundance of *Pseudobutyrivibrio*, but greater abundance of *Succinivibrionaceae UCG-002*, *unclassified genus in Bacteroidetes*, and *Succinivibrio*, than LSO-fed kids. The relative abundances of the 20 most abundant bacteria (family level) showed that MIX-fed kids had a lower abundance of Acidaminococcaceae and Veillonellaceae, but greater abundance of Bacteroidales_S24–7_group, than LSO-fed kids. All of these outcomes would probably lead to a decrease in the proportional loss rate of C18:3n-3 and an increase in the proportional loss rate of *cis-*9 C18:1 in MIX-fed kids compared with LSO-fed kids, as observed *in vitro* [6].

Increases in C18:0 are a consequence of the extensive biohydrogenation in the goat rumen [5]. The abundance of *Ruminococcus_2* was positively correlated with the proportional increment rate in C18:0 in the present study, which indicated that *Ruminococcus_2* is related to hydrogenation, consistent with the ability of H_2_ production [39]. MIX-fed kids had the highest abundance of *Ruminococcus_2*, consistent with the high proportion of rumen C18:0 in MIX-fed kids. However, PMO-fed kids had the lowest abundance of *Ruminococcus_2*, but the highest proportional increment rate in C18:0. The implication is that PMO-kids had a high abundance of other bacteria that are responsible for the proportional increment rate of C18:0. The inclusion of different oils in the diet did not influence bacterial richness or diversity, as reported by other laboratories [40], with Bacteroidetes, Firmicutes, Proteobacteria, and Verrucomicrobia remaining the dominant phyla for all diets, as seen in other studies with goats and sheep [5, 40].

The current study used proportional loss rate of FA to reflect FA biohydrogentation, and proportional increment rate in FA to reflect FA synthesis. Future research should consider more accurate calculation of the hydrogenation of FA and synthesis of FA, the passage rate, rumen volume, intake of FA, and rumen FA concentration.

### Mixed oil decreases the expression of genes related to FA oxidation and increases n-3 LCPUFA content in muscle

The key enzyme involved in oxidation of FA is *CPT1β* [41] and the most relevant enzymes in n-3 LCPUFA biosynthesis are *FADS1* and *FADS2* [10]. In the present study, the MIX diet decreased mRNA expression for *CPT1β* in muscle, compared with the LSO diet, thereby probably decreasing the production of the enzyme and the oxidation of C18:3n-3, helping explain the increase in the muscle concentration of C18:3n-3. Moreover, if less C18:3n-3 is oxidized, more substrate would be made available for n-3 LCPUFA synthesis, and up-regulation of mRNA expression of *FADS1* and *FADS2* would be observed, leading to an increase in the conversion of C18:3n-3 to C20:5n-3 and C22:6n-3.

Our observations are also consistent with previous studies in the goat, where a linseed oil supplement increased mRNA expression of *CPT1β* in adipose tissue [16], and in the rabbit where mRNA expression of *CPT1* in liver increased as dietary C18:3n-3 content increased [42], and palm oil decreased the TBA-reactive substances thereby improving the lipid stability of PUFA-modified animal products (meat, egg and liver) [43].Taking all these findings into account, we conclude that combining linseed oil with palm oil offers a new approach for increasing the content of C18:3n-3 in goat meat, and oils with a high content of PUFA can be stablised *in vivo* by blending them with oils of high *cis-*9 C18:1 content, as originally suggested by Emmison et al. [11].

### The effects of different dietary oils on the predominant FAs in goat muscle

We found the predominant FAs in goat muscle to be C16:0 and C18:0 as SFAs, *cis-*9 C18:1 as MUFAs, and C18:2n-6*c* as PUFAs, as reported by Ebrahimi et al. [3]. Profiles of C16:0 in the rumen, plasma, and liver also reflect the muscle profiles, with the diets ranking PMO ≥ MIX ≥ LSO, an observation from another study (Wang et al., unpublished), although muscle values did not differ significantly among these three treatments in the present study. Annison and Bryden [44] suggested that tissue C16:0 is derived from a combination of *de novo* synthesis and extraction from circulating plasma. Fatty acid synthetase (*FAS*) plays a central role in *de novo* lipogenesis in animals by catalyzing all the reactions involved in the conversion of acetyl-coA and malonyl-CoA to palmitate [45]. Increased mRNA expression of *FAS* in the muscle of LSO-fed and MIX-fed kids is assumed to increase the C16:0 concentration in muscle, thus nullifying the effect of the diet and explaining the lack of difference among the three groups in the muscle concentration of C16:0. In addition, *ELOVL6* activity elongates C16:0 and *cis*-9 C16:1 to C18:0 and *cis*-9 C18:1 [46], whereas *SCD* desaturates C18:0 to *cis*-9 C18:1 [47]. The results of the present study suggest that the lack of difference in mRNA expression of *ELOVL6* and *SCD1* in muscle among the three groups would explain the similar concentrations of C16:1, C18:0, and *cis-*9 C18:1. Again, these observations agree with others, using various tissues, showing that linseed oil does not affect *SCD1*, *ACC* or *ELOVL6* [48, 49], but increases the mRNA expression of *FAS* [9, 50].

### Difference in FA composition between muscle types

In sheep, *Biceps femoris* typically has a lower lipid content than *Longissimus dorsi* whereas a muscle containing low concentrations of lipid would have a greater proportion of functional FA, such as PUFA [51, 52]. These observations explain why the concentration of PUFA was greater in *Biceps femoris* than in *Longissimus dorsi* in the present study. Working with bovine muscle, Talmant et al. [53] found that *Longissimus dorsi* has a greater glycolytic activity than *Biceps femoris*, implying greater production of ATP, an essential driver of *de novo* synthesis of SFA. In the present study, SFA concentration was greater in *Longissimus dorsi* than in *Biceps femoris*, and there was proportionately less n-6 PUFA in *Longissimus dorsi* than in *Biceps femoris*. These observations suggest that a greater glycolytic activity and a lesser mitochondrial oxidative activity lead to a lower n-6/n-3 ratio in *Longissimus dorsi* compared with *Biceps femoris*. This hypothesis is supported by the observation in cattle that *Biceps femoris* has a greater content of PUFA, P/S, and n-6/n-3 ratios, but a lesser content of SFA, compared with *Longissimus dorsi* [52].

### The interaction between oil type and muscle on n-3 PUFAs and n-6 PUFAs

We observed that C18:3n-3 concentration was affected by both oil type and muscle type, with a lower concentration in both muscles with PMO treatment and a higher concentration in *Biceps femoris* with all oil treatments (expect for PMO). These observations agree with other studies showing that C18:3n-3 concentration is greater in *Biceps femoris* than in *Longissimus dorsi* in goats [54], and that dietary palm oil decreases C18:3n-3 concentrations in liver, adipose, and muscle of piglets, compared with linseed oil [55]. In mammals, C18:3n-3 is the subtrate for the synthesis of C22:6n-3 [4]. In *Biceps femoris* with both linseed-based treatments (LSO and MIX), the C18:3n-3 concentration was greater than in *Longissimus dorsi* with any treatment but, for C22:6n-3 concentration, the opposite outcome was observed. These observations suggest that *Biceps femoris* has a poor ability to synthesise C22:6n-3 from C18:3n-3 compared with *Longissimus dorsi*, as previously reported for goat meat [54]. Studies also demonstrated a higher C18:2n-6 content in *Biceps femoris* than in *Longissimus dorsi* [54, 56]. In the present study, we observed a higher C18:2n-6*t* concentratio in *Biceps femoris* than in *Longissimus dorsi,* independently of oil treatment, leading to a higher n-6/n-3 ratio in *Biceps femoris* than in *Longissimus dorsi*.

## Conclusions

Feeding a combination of linseed and palm oils (ratio of 2:1, weight:weight) to cashmere goat kids is an efficient method for increasing the muscle concentrations of C18:3n-3, C20:5n-3, C22:6n-3, and n-3 LCPUFA, and for decreasing the muscle n-6/n-3 ratio.

## Supplementary information

**Additional file 1: Table S1.** Primer pairs sequences for quantitative real-time PCR.

**Additional file 2: Figure S1.** The OTU rarefaction curves of the ruminal digesta bacterial communities. Curves were drawn using the least sequenced sample as upper limit for the rarefactions. Each color represents a dietary treatment: PMO (red); LSO (blue); MIX (green).

**Additional file 3: Figure S2.** The approximately-maximum-likelihood phylogenetic trees revealed that *unclassified_k_norank* clustered within the *Bacteroidetes* phylum (constructed using FastTree in R, version 2.1.3 http://www.microbesonline.org/fasttree/).

**Additional file 4: Figure S3.** Relative abundance of various communities of bacteria (phylum level) in the rumen of goat kids fed the palm oil (P1-P6), linseed oil (L1-L6) and mixed oil (M1-M6) diets.

## Data Availability

The raw data for the current study are available from the corresponding author on reasonable request.

## Abbreviations

n-3 LCPUFA: n-3 poly-unsaturated fatty acids
FA: Fatty acid
nMDS: Non-metric multidimensional scaling
SFA: Saturated fatty acids
MUFA: Monounsaturated fatty acids
n-6/n-3: n-6 polyunsaturated fatty acid/n-3 polyunsaturated fatty acid ratio
U/S: Unsaturated fatty acid/saturated fatty acid ratio
P/S: Polyunsaturated fatty acid/saturated fatty acid ratio
FAS: Fatty acid synthetase
ACC: Acetyl-CoA carboxylase
SCD1: Stearoyl-CoA desaturase 1
FADS1: Delta-5 desaturase
FADS2: Delta-6 desaturase
ELVOL5: Elongation of very long chain fatty acids protein 5
ELOVL6: Elongation of very long chain fatty acids protein 6
ACOX1: Acyl-coenzyme A oxidase 1
CPT1β: Carnitine palmitoyltransferase I
TMR: Total mixed ration

## References

[1] Kromhout D. Fish (oil) consumption and coronary heart disease. In book: Dietary ω3 and ω6 Fatty Acids. 1989;273–282. doi:10.1007/978-1-4757-2043-3_25.

[2] Barlow SM, Young FVK, Duthie IF (1990). Nutritional recommendations for n-3 polyunsaturated fatty acids and the challenge to the food industry. Proc Nutr Soc.

[3] Ebrahimi M, Rajion MA, Goh YM (2014). Effects of oils rich in linoleic and α-linolenic acids on fatty acid profile and gene expression in goat meat. Nutrients..

[4] Gruffat D, Cherfaoui M, Bonnet M, Thomas A, Bauchart D, Durand D (2013). Breed and dietary linseed affect gene expression of enzymes and transcription factors involved in n-3 long chain polyunsaturated fatty acids synthesis in longissimus thoracis muscle of bulls. J Anim Sci.

[5] Cremonesi P, Conte G, Severgnini M, Turri F, Monni A, Capra E (2018). Evaluation of the effects of different diets on microbiome diversity and fatty acid composition of rumen liquor in dairy goat. Animal..

[6] Adeyemi KD, Ebrahimi M, Samsudin AA, Alimon AR, Karim R, Karsani SA (2015). Influence of Carotino oil on in vitro rumen fermentation, metabolism and apparent biohydrogenation of fatty acids. Anim Sci J.

[7] Gruffat D, Gobert M, Durand D, Bauchart D (2011). Distinct metabolism of linoleic and linolenic acids in liver and adipose tissues of finishing Normande cull cows. Animal..

[8] González-Ortiz G, Sala R, Cánovas E, Abed N, Barroeta AC (2013). Consumption of dietary n-3 fatty acids decreases fat deposition and adipocyte size, but increases oxidative susceptibility in broiler chickens. Lipids..

[9] Li WH, Tang DF, Li FD, Tian HQ, Yue XP, Li F (2017). Supplementation with dietary linseed oil during peri-puberty stimulates steroidogenesis and testis development in rams. Theriogenology..

[10] Valenzuela R, Echeverria F, Ortiz M, Rincón-Cervera MÁ, Espinosa A, Hernandez-Rodas MC (2017). Hydroxytyrosol prevents reduction in liver activity of Δ-5 and Δ-6 desaturases, oxidative stress, and depletion in long chain polyunsaturated fatty acid content in different tissues of high-fat diet fed mice. Lipids Health Dis.

[11] Emmison N, Gallagher PA, Coleman RA (1995). Linoleic and linolenic acids are selectively secreted in triacylglycerol by hepatocytes from neonatal rats. Am J Physiol-Reg I.

[12] China NY/T816. Feeding standard of meat-producing sheep and goats. China NongYe HangYe Biaozhun/Tuijian-816. China Agricultural Publisher, Beijing, China. 2004.

[13] AOAC. Official methods of analysis. 17th ed. Assoc. Off. Anal. Chem., Arlington, VA. 2002.

[14] Van Soest PJ, Robertson JB, Lewis BA (1991). Methods for dietary fiber, neutral detergent fiber, and non-starch polysaccharides in relation to animal nutrition. J Dairy Sci.

[15] O'Fallon JV, Busboom JR, Nelson ML, Gaskins CT (2007). A direct method for fatty acid methyl ester synthesis: application to wet meat tissues, oils, and feedstuffs. J Anim Sci.

[16] Wang X, Martin GB, Liu S, Shi B, Guo X, Zhao Y, et al. The mechanism through which dietary supplementation with heated linseed grain increases n-3 long-chain polyunsaturated fatty acid concentration in subcutaneous adipose tissue of cashmere kids. J Anim Sci. 2018. 10.1093/jas/sky386. 10.1093/jas/sky386 PMC6313143 30312437

[17] Wang X, Wu TM, Yan SM, Shi BL, Zhang Y, Guo XY (2019). Influence of pasture or total mixed ration on fatty acid composition and expression of lipogenic genes of longissimus thoracis and subcutaneous adipose tissues in Albas White Cashmere Goats. Italian J Anim Sci.

[18] Livak KJ, Schmittgen TD (2001). Analysis of relative gene expression data using real-time quantitative PCR and the 2−ΔΔCT method. Methods..

[19] Vandesompele J, De Preter K, Pattyn F, Poppe B, Van Roy N, De Paepe A, et al. Accurate normalization of real-time quantitative RT-PCR data by geometric averaging of multiple internal control genes. Genome Biol. 2002;3:research0034–1. doi:10.1186/gb-2002-3-7-research0034. 10.1186/gb-2002-3-7-research0034 PMC126239 12184808

[20] Evans CC, Lepard KJ, Kwak JW, Stancukas MC, Laskowski S, Dougherty J (2014). Exercise prevents weight gain and alters the gut microbiotaina mouse model of high fat diet-induced obesity. PLoS One.

[21] Caporaso JG, Kuczynski J, Stombaugh J, Bittinger K, Bushman FD, Costello EK (2010). QIIME allows analysis of high-throughput community sequencing data. Nat Methods.

[22] Meale SJ, Ding S, He ML, Dugan MER, Ribeiro GO, Alazzeh AY, Holo H, Harstad OM, McAllister TA, Chaves AV (2014). Effect of Propionibacterium freudenreichii, on ruminal fermentation patterns, methane production and lipid biohydrogenation of beef finishing diets containing flaxseed oil in a rumen simulation technique. Can J Anim Sci.

[23] Oliveira MA, Alves SP, Santos-Silva J, Bessa RJB (2016). Effects of clays used as oil adsorbents in lamb diets on fatty acid composition of abomasal digesta and meat. Anim Feed Sci Technol.

[24] Alves SP, Francisco A, Costa M, Santos-Silva J, Bessa RJB (2017). Biohydrogenation patterns in digestive contents and plasma of lambs fed increasing levels of a tanniferous bush (*Cistus ladanifer* L.) and vegetable oils. Animal Feed Sci Technol.

[25] Ebrahimi M, Rajion MA, Goh YM, Sazili AQ, Schonewille JT (2013). Effect of linseed oil dietary supplementation on fatty acid composition and gene expression in adipose tissue of growing goats. Biomed Res Int.

[26] Noosen P, Lounglawan P, Suksombat W (2017). Linseed oil supplemented concentrate fed to Brahman crossbred fattening steers on carcass quality traits and intramuscular fatty acid profiles. Songklanakarin J Sci Technol.

[27] Cepeljnik T , Devillard E . Capability of biohydrogenation of linoleic acid in rumen bacterium Pseudobutyrivibrio xylanivorans Mz5T. Acta Agriculturae Slovenica; 2006;88:75–81.

[28] Benz J, Wolf C, Rüdiger W (1980). Chlorophyll biosynthesis: hydrogenation of geranylgeraniol. Plant Science Letters.

[29] Rosenfeld IS, Tove SB (1971). Biohydrogenation of unsaturated fatty acids VI source of hydrogen and stereospecifity of reduction. J Biol Chem.

[30] Hughes PE, Tove SB (1980). Identification of an endogenous electron donor for biohydrogenation as alpha-tocopherolquinol. J Biol Chem.

[31] Wolin MJ, Miller TL, Stewart CS. Microbe-microbe interactions. The rumen microbial ecosystem. 1997;467–491. doi: 10.1007/978-1-4757-0322-1_10.

[32] Santos EDO, Thompson F. The family succinivibrionaceae. The Prokaryotes. Springer Berlin Heidelberg. 2014;639–648. doi:10.1007/0-387-30743-5_20.

[33] Scully ED, Geib SM, Carlson JE, Tien M, McKenna D, Hoover K (2014). Functional genomics and microbiome profiling of the Asian longhorned beetle (Anoplophora glabripennis) reveal insights into the digestive physiology and nutritional ecology of wood feeding beetles. BMC Genomics.

[34] Chen H, Zhao R, Wang B, Zheng L, Ouyang H, Wang H (2018). Acute oral administration of single-walled carbon nanotubes increases intestinal permeability and inflammatory responses: association with the changes in gut microbiota in mice. Adv Healthc Mater.

[35] Gagen EJ, Padmanabha J, Denman SE, Christopher SM (2015). Hydrogenotrophic culture enrichment reveals rumen Lachnospiraceae and Ruminococcaceae acetogens and hydrogen-responsive Bacteroidetes from pasture-fed cattle. FEMS Microbiol Lett.

[36] Petri RM, Vahmani P, Yang HE, Dugan ME, McAllister TA. Changes in rumen microbial profiles and subcutaneous fat composition when feeding extruded flaxseed mixed with or before hay. Front Microbiol. 2018;9. 10.3389/fmicb.2018.01055. 10.3389/fmicb.2018.01055 PMC5981202 29887841

[37] Wang X, Martin GB, Wen Q (2019). Linseed oil and heated linseed grain supplements have different effects on rumen bacterial community structures and fatty acid profiles in cashmere kids1. J Anim Sci.

[38] Vries de W, Rietveld-Struijk TRM, Stouthamer AH. ATP formation associated with fumarate and nitrate reduction in growing cultures of Veillonella alcalescens. Anton Leeuw Int J G 1977;43:153–167. doi:10.1007/BF00395670. 10.1007/BF00395670 202192

[39] Chaucheyras-Durand F, Masséglia S, Fonty G, Forano E (2010). Influence of the composition of the cellulolytic flora on the development of hydrogenotrophic microorganisms, hydrogen utilization, and methane production in the rumens of gnotobiotically reared lambs. Appl Environ Microbiol.

[40] Lyons T, Boland T, Storey S, Doyle E (2017). Linseed oil supplementation of kids’ diet in early life leads to persistent changes in rumen microbiome structure. Front Microbiol.

[41] Paumen MB, Ishida Y, Muramatsu M, Yamamoto M, Honjo T (1997). Inhibition of carnitine palmitoyltransferase I augments sphingolipid synthesis and palmitate-induced apoptosis. J Biol Chem.

[42] Du HT, Wang CY, Wang XP, Ma MW, Li FC (2013). The effects of dietary α-linolenic acid on growth performance, meat quality, fatty acid composition, and liver relative enzyme mRNA expression of growing meat rabbits. J. Anim. Feed Sci.

[43] Kang KR, Cherian G, Sim JS (2001). Dietary palm oil alters the lipid stability of polyunsaturated fatty acid-modified poultry products. Poult Sci.

[44] Annison EF, Bryden WL (1999). Perspectives on ruminant nutrition and metabolism. II. Metabolism in ruminant tissues. Nutr. Res. Rev..

[45] Weiss GH, Rosen OM, Rubin CS (1980). Regulation of fatty acid synthetase concentration and activity during adipocyte differentiation. Studies on 3T3-L1 cells. J Bio Chem.

[46] Jakobsson A, Westerberg R, Jacobsson A (2006). Fatty acid elongases in mammals: their regulation and roles in metabolism. Prog Lipid Res.

[47] Vernon RG (1980). Lipid metabolism in the adipose tissue of ruminant animals. Prog Lipid Res.

[48] Faulconnier Y, Bernard L, Boby C, Domagalski J, Chilliard Y, Leroux C (2018). Extruded linseed alone or in combination with fish oil modifies mammary gene expression profiles in lactating goats. Animal..

[49] Chen K, Li E, Xu C, Wang X, Lin H, Qin JG, Chen L (2015). Evaluation of different lipid sources in diet of pacific white shrimp Litopenaeus vannamei at low salinity. Aquaculture Reports.

[50] Kim JS, Ingale S, Lee SH, Choi YH, Kim EH, Lee DC (2014). Impact of dietary fat sources and feeding level on adipose tissue fatty acids composition and lipid metabolism related gene expression in finisher pigs. Anim Feed Sci Technol.

[51] Erkigul B, Gerelt B, Damdinsuren L (2018). The seasonal effects on IMF content of FAS mRNA expression in muscles of Mongolian sheep. Mongolian J Agricultural Sci.

[52] Wolf C, Ulbrich SE, Kreuzer M, Berard J, Giller K (2018). Differential partitioning of rumen-protected n-3 and n-6 fatty acids into muscles with different metabolism. Meat Sci.

[53] Talmant A, Monin G, Briand M, Dadet M, Briand Y (1986). Activities of metabolic and contractile enzymes in 18 bovine muscles. Meat Sci.

[54] Kiani A, Fallah R (2016). Effects of live weight at slaughter on fatty acid composition of Longissimus dorsi and biceps femoris muscles of indigenous Lori goat. Trop Anim Health Prod.

[55] Missotten J, De Smet S, Raes K, Doran O (2009). Effect of supplementation of the maternal diet with fish oil or linseed oil on fatty-acid composition and expression of Δ5- and Δ6-desaturase in tissues of female piglets. Animal..

[56] Sharma N, Gandemer G, Goutefongea R, Kowale BN (1986). Fatty acid composition of water buffalo meat. Meat Sci.

