# Supplementary material for: Palm oil protects α-linolenic acid from rumen biohydrogenation and muscle oxidation in cashmere goat kids
Source: J Anim Sci Biotechnol. 2020 Oct 5;11:100. doi: 10.1186/s40104-020-00502-w (PMC7534170; doi:10.1186/s40104-020-00502-w)
Supplement: Supplementary file 1 — Additional file 1: Table S1. Primer pairs sequences for quantitative real-time PCR. [file 40104_2020_502_MOESM1_ESM.docx]

| **Supplementary Table 1.** Primer pairs sequences for quantitative real-time PCR. | | | | | | |
| --- | --- | --- | --- | --- | --- | --- |
| **Gene^1^** | **Primer pairs(5’→3’)** | **Accessions number** | **Annealing temperature, °C** | **Length, bp** | **Primer efficiency** | **R^2^** |
| *FADS1* | F:ATGCAACGTCCACAAGTCAG | XM_004019593 | 56 | 115 | 1.90 | 0.98 |
|  | R:GGGAGCCACTTTGTGGTAAT |  |  |  |  |  |
| *FADS2* | F:GTTCAGTGGGCACCTCAACT | XM_004019592 | 57 | 106 | 2.00 | 0.99 |
|  | R:TACTCAATGCCGTGCTTGG |  |  |  |  |  |
| *ELOVL5* | F:GGAAGGCCGGTACAACTTCT | XM_004018905 | 56 | 118 | 1.99 | 0.99 |
|  | R:TGTCCATGAACTCGATGAGC |  |  |  |  |  |
| *CPT1**β* | F:TGTTCAACACCACTCGCATC | AJ272435 | 58 | 116 | 1.97 | 0.98 |
|  | R:CTCGTAGAGCCACAGCTTGA |  |  |  |  |  |
| *ELOVL6* | F:CTCTGGTCTCTGACCCTTGC | XM_004009618 | 57 | 89 | 1.96 | 0.98 |
|  | R:AGGCCTTTGGTCATCACAGT |  |  |  |  |  |
| *ACOX1* | F:GAGTGAGCTGCCTGAGCTTC | NM_001035289 | 59 | 62 | 1.96 | 0.98 |
|  | R:TTGTCCAGGACGTGAAAGC |  |  |  |  |  |
| *SCD1* | F:CCCAGCTGTCAGAGAAAAGG | AJ001048 | 60 | 115 | 1.96 | 0.98 |
|  | R:GATGAAGCACAACAGCAGGA |  |  |  |  |  |
| *ACC* | F:ACCCAACCCAGAAAGGTCAGT | NM_001009256 | 60 | 125 | 1.95 | 0.98 |
|  | R:TCCCACGGGTATTCCTCCTA |  |  |  |  |  |
| *FAS* | F:GCAACCAGGGGAGACCGT | AB011671 | 60 | 300 | 1.99 | 0.99 |
|  | R:CTGAGGGCAATGGCGATGG |  |  |  |  |  |
| *β-actin* | F:ACTGGGACGACATGGAGAAGA | U39357 | 60 | 199 | 2.00 | 0.99 |
|  | R:GCGTACAGGGACAGCACAG |  |  |  |  |  |
| *β2M* | F:GGTGCTGCTTAGAGGTCTCG | NM_001009284 | 59 | 109 | 1.99 | 0.99 |
|  | R:ACGCTGAGTTCACTCCCAAC |  |  |  |  |  |
| *YWHAZ* | F:TGTAGGAGCCCGTAGGTCATCT | AY970970 | 59 | 102 | 1.99 | 0.99 |
|  | R:TTCTCTCTGTATTCTCGAGCCATCT |  |  |  |  |  |

^1^ *FAS* = fatty acid synthetase, *ACC* = acetyl-CoA carboxylase, *SCD1* = stearoyl-CoA desaturase 1, *FADS1* = delta-5 desaturase, *FADS2* = delta-6 desaturase, *ELVOL5* = elongation of very long chain fatty acids protein 5, *ELOVL6* = elongation of very long chain fatty acids protein 6, *ACOX1* = acyl-coenzyme A oxidase 1, *CPT1β* = carnitine palmitoyltransferase I, *β2M* = β-2-microglobulin, *YWHAZ* = tyrosine 3-monooxygenase, *β-actin* = beta-actin
