# Supplementary material for: Palm oil protects α-linolenic acid from rumen biohydrogenation and muscle oxidation in cashmere goat kids
Source: J Anim Sci Biotechnol. 2020 Oct 5;11:100. doi: 10.1186/s40104-020-00502-w (PMC7534170; doi:10.1186/s40104-020-00502-w)
Supplement: Supplementary file 2 — Additional file 2: Figure S1. The OTU rarefaction curves of the ruminal digesta bacterial communities. Curves were drawn using the least sequenced sample as upper limit for the rarefactions. Each color represents a dietary treatment: PMO (red); LSO (blue); MIX (green). [file 40104_2020_502_MOESM2_ESM.docx]

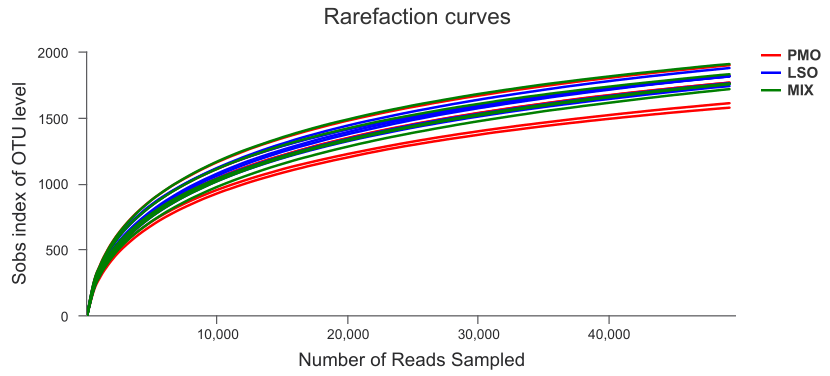


**Supplementary Fig. 1** The OTU rarefaction curves of the ruminal digesta bacterial communities. Curves were drawn using the least sequenced sample as upper limit for the rarefactions. Each color represents a dietary treatment: PMO (red); LSO (blue); MIX (green).
