# Supplementary material for: Palm oil protects α-linolenic acid from rumen biohydrogenation and muscle oxidation in cashmere goat kids
Source: J Anim Sci Biotechnol. 2020 Oct 5;11:100. doi: 10.1186/s40104-020-00502-w (PMC7534170; doi:10.1186/s40104-020-00502-w)
Supplement: Supplementary file 3 — Additional file 3: Figure S2. The approximately-maximum-likelihood phylogenetic trees revealed that unclassified_k_norank clustered within the Bacteroidetes phylum (constructed using FastTree in R, version 2.1.3 http://www.microbesonline.org/fasttree/). [file 40104_2020_502_MOESM3_ESM.docx]

**Supplementary Fig. 2** The approximately-maximum-likelihood phylogenetic trees revealed that *unclassified_k_norank* clustered within the *Bacteroidetes* phylum (constructed using FastTree in R, version 2.1.3 http://www.microbesonline.org/fasttree/).
