# Supplementary material for: Palm oil protects α-linolenic acid from rumen biohydrogenation and muscle oxidation in cashmere goat kids
Source: J Anim Sci Biotechnol. 2020 Oct 5;11:100. doi: 10.1186/s40104-020-00502-w (PMC7534170; doi:10.1186/s40104-020-00502-w)
Supplement: Supplementary file 4 — Additional file 4: Figure S3. Relative abundance of various communities of bacteria (phylum level) in the rumen of goat kids fed the palm oil (P1-P6), linseed oil (L1-L6) and mixed oil (M1-M6) diets. [file 40104_2020_502_MOESM4_ESM.docx]

**Supplementary Fig. 3** Relative abundance of various communities of bacteria (phylum level) in the rumen of goat kids fed the palm oil (P1-P6), linseed oil (L1-L6) and mixed oil (M1-M6) diets.
